# The impact of Paclitaxel-based hyperthermic intraperitoneal chemotherapy in advanced high-grade serous ovarian cancer patients - interim analysis of safety and immediate efficacy of a randomized control trial (C-HOC trial)

**DOI:** 10.1186/s13048-024-01468-3

**Published:** 2024-07-12

**Authors:** Qun Wang, Hua Liu, Yuhong Shen, Lifei Shen, Jian Li, Weiwei Feng

**Affiliations:** 1 Department of Gynecology and Obstetrics, Ruijin Hospital, Shanghai Jiao Tong University School of Medicine, Shanghai, China; 2 Clinical Research Center, Ruijin Hospital, Shanghai Jiao Tong University School of Medicine, Shanghai, China

**Keywords:** High-grade serous ovarian/Fallopian tube carcinoma (HGSOC), Paclitaxel, Neoadjuvant chemotherapy, Hyperthermic intraperitoneal chemotherapy (HIPEC), Chemotherapy response scores (CRS)

## Abstract

**Objective:**

This study evaluates the potential superiority of combining paclitaxel-based hyperthermic intraperitoneal chemotherapy (HIPEC) with sequential intravenous neoadjuvant chemotherapy over intravenous neoadjuvant chemotherapy alone in Chinese patients with Federation of Gynecology and Obstetrics (FIGO) stage IIIC, IVA and IVB high-grade serous ovarian/fallopian tube carcinoma (HGSOC). This interim analysis focuses on the safety and immediate efficacy of both regimens to determine the feasibility of the planned trial (C-HOC Trial).

**Methods:**

In a single-center, open-label, randomized control trial, FIGO stage IIIC, IVA, and IVB HGSOC patients (FAGOTTI score ≥ 8 during laparoscopic exploration) unsuitable for optimal cytoreduction in primary debulking surgery (PDS) were randomized 2:1 during laparoscopic exploration. The Experiment Group (HIPEC Group) received one cycle of intraperitoneal neoadjuvant laparoscopic hyperthermic intraperitoneal chemotherapy (paclitaxel) followed by three cycles of intravenous chemotherapy (paclitaxel plus carboplatin), while the Control Group received only three cycles of intravenous chemotherapy. Both groups subsequently underwent interval debulking surgery (IDS). The adverse effects of chemotherapy, postoperative complications, and pathological chemotherapy response scores (CRS) after IDS were compared.

**Results:**

Among 65 enrolled patients, 39 HIPEC Group and 21 Control Group patients underwent IDS. Grade 3–4 chemotherapy-related adverse effects were primarily hematological with no significant differences between the two groups. The HIPEC Group exhibited a higher proportion of CRS 3 (20.5% vs. 4.8%; *P* = 0.000). R0 resection rates in IDS were 69.2% (HIPEC Group) and 66.7% (Control Group). R2 resection occurred in 2.6% (HIPEC Group) and 14.3% (Control Group) cases. No reoperations or postoperative deaths were reported, and complications were managed conservatively.

**Conclusions:**

Combining HIPEC with IV NACT in treating ovarian cancer demonstrated safety and feasibility, with no increased chemotherapy-related adverse effects or postoperative complications. HIPEC improved tumor response to neoadjuvant chemotherapy, potentially enhancing progression-free survival (PFS). However, the final overall survival results are pending, determining if HIPEC combined with IV NACT is superior to IV NACT alone.

## Introduction

Ovarian cancer stands as the gynecologic malignancy with the highest mortality rate. Despite significant advances in targeted and immunotherapy in recent years, the overall 5-year survival rate remains below 50% [1]. High-grade serous cancer (HGSOC) predominates in this category [2]. HGSOC typically presents as asymptomatic and is challenging to diagnose at an early stage. Approximately 75% of HGSOC patients are diagnosed with advanced disease (FIGO IIIC, IVA, and IVB stages), contributing to over 70% of all ovarian cancer-related deaths [2, 3].

Intraperitoneal dissemination serves as the primary mode of advanced HGSOC metastasis and is a key factor in treatment failure and recurrence [2]. There is evidence suggesting that combining intravenous and intraperitoneal chemotherapy can enhance and prolong patient survival [4, 5]. Nevertheless, the widespread adoption of this approach is impeded by catheter-related issues and the severe toxic side effects associated with intraperitoneal chemotherapy.

Intraperitoneal hyperthermic chemotherapy (HIPEC) represents an improved approach to intraperitoneal chemotherapy, and it has been employed in clinical practice for decades. The well-documented OVHIPEC-01 study validated the use of HIPEC in conjunction with interval debulking surgery (IDS) for enhancing the prognosis of patients with advanced epithelial ovarian cancer [6]. However, the effectiveness of HIPEC in the context of neoadjuvant chemotherapy remains uncertain. Recent retrospective studies have indicated that HIPEC can enhance the chemotherapy response scores (CRS) and reduce the recurrence rate among patients with advanced high-grade serous ovarian cancer [7]. Nevertheless, randomized trial data are currently lacking.

To address this knowledge gap, we conducted a single-center, open-label randomized controlled Trial: The Impact of Paclitaxel-Based Hyperthermic Intraperitoneal Chemotherapy (HIPEC) Followed by Sequential Intravenous Chemotherapy in Advanced High-Grade Serous Ovarian Cancer Patients – HIPEC for Ovarian Cancer in China (C-HOC Trial). We aimed to investigate whether paclitaxel-based HIPEC, when combined with intravenous neoadjuvant chemotherapy, offers an advantage over intravenous neoadjuvant chemotherapy alone in improving the NACT response score in patients with advanced HGSOC. Additionally, we explored whether the inclusion of HIPEC in neoadjuvant therapy led to increased adverse reactions and had a negative impact on IDS outcomes.

The primary endpoint of this trial was the difference in overall survival between the two groups. For this interim analysis, we compared the adverse effects of chemotherapy and postoperative complication rates after IDS between the two groups. Additionally, we assessed the immediate treatment efficacy by comparing the rate of pathological chemotherapy response scores (CRS) after IDS.

### Trial design

**Fig. 1:**
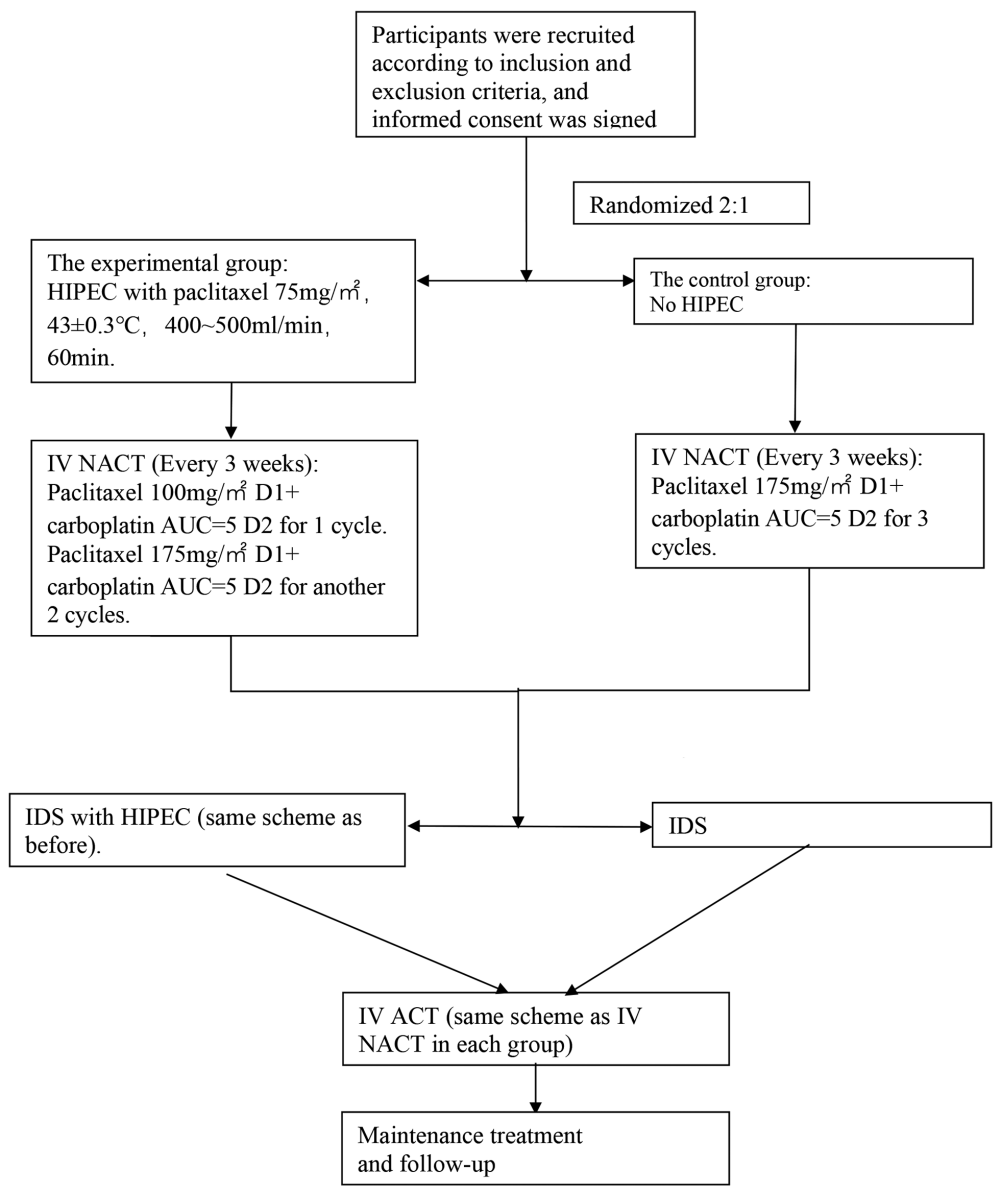
The study schema

In a single-center, open-label, randomized control trial(Fig. 1 ), patients with FIGO stage IIIC, IVA and IVB HGSOC, who were evaluated with a FAGOTTI score ≥ 8 during laparoscopic exploration and were unable to undergo optimal cytoreduction (no visible disease (R0) or one or more residual tumors measuring 10 mm or less in diameter (R1) resection) in primary debulking surgery (PDS), were randomized into two groups in a 2:1 ratio. Randomization occurred at the time of laparoscopic exploration.

### Participants

#### Inclusion criteria

This study encompasses newly diagnosed ovarian cancer patients falling within the age range of 18 to 75 years, exhibiting an Eastern Cooperative Oncology Group (ECOG) performance status score between 0 and 2. Included participants must not have undergone any prior anti-tumor therapies, including radiotherapy, chemotherapy, or targeted therapy. Eligible patients should have undergone a preoperative examination coupled with intraoperative exploration and evaluation, resulting in a diagnosis of International Federation of Gynecology and Obstetrics (FIGO) stage IIIC, IVA, and IVB ovarian cancer. Additionally, participants must have attained a FAGOTTI score of at least 8 during laparoscopy, confirming the presence of High-Grade Serous Ovarian Cancer (HSGOC) through rapid pathology assessment. Moreover, individuals included in the study must exhibit sufficient bone marrow reserves and normal organ function, characterized by white blood cell counts of ≥ 3.5 × 10^9/L, neutrophil counts of ≥ 1.5 × 10^9/L, hemoglobin levels of ≥ 80 g/L, and platelet counts of ≥ 80.0 × 10^9/L. Serum bilirubin, alanine aminotransferase (ALT), and aspartate aminotransferase (AST) levels should all be within the upper limits of normal. Likewise, urea nitrogen (BUN) and creatinine (Cr) levels should not exceed the upper limits of normal. Lastly, prospective participants must provide written informed consent to be included in the study.

#### Exclusion criteria

This study excludes individuals with serious or uncontrolled medical and surgical conditions or acute infections. Pregnant or breastfeeding female patients are also ineligible for participation. Additionally, individuals with a history of gastrointestinal bleeding, perforation, intestinal obstruction, or related diseases are not included in this study.

#### Reasons for Dropout

Patients who were enrolled in the study were subject to dropout if they failed to adhere to the prescribed study protocol or voluntarily withdrew their consent for any personal reasons. Additionally, participants were removed from the study if they became unable to complete the planned treatment for any unforeseen circumstances or if they declined to undergo surgery at the same hospital as per the study requirements.

### Data collection

The study was carried out and analyzed under the auspices of the Department of Obstetrics and Gynecology at Ruijin Hospital. Oversight and monitoring of the study were conducted by the Clinical Research Center of Ruijin Hospital, the official body responsible for guiding and supervising various research endeavors within the hospital. Timely meetings were held to ensure adherence to protocol guidelines throughout the study’s implementation. This study received approval from the Ethics Committee of Ruijin Hospital and was registered on the Chinese Clinical Trial Registry platform (ChiCTR2000028894). Informed consent was obtained from all enrolled patients, beginning on September 2, 2019.

### Interventions

#### Laparoscopic exploration

Patients underwent laparoscopic exploration under general anesthesia. Ascites, if present, were aspirated and measured. Suspected primary lesions or metastatic lesions were excised at a minimum of 2 points and sent for rapid pathology. FAGOTTI and Peritoneal Cancer Index scores (PCI) were calculated based on the exploration results.

#### HIPEC Treatment

After completing the initial endoscopic exploration, the experimental group underwent immediate HIPEC treatment under intraoperative general anesthesia. HIPEC was administered using the body cavity hyperthermic perfusion therapy system (Guangzhou Borui Medical Technology Co. LTDBR-TRG-II type) with paclitaxel 75 mg/m², normal salin4000 ml, at a flow rate of 400–500 mL/min, and a temperature of 43 ± 0.3 °C for 60 min. The indwelling tubes included 2 inflow tubes and 2 outflow tubes. Temperature monitoring probes in both the inflow and outflow tubes ensured real-time intra-abdominal temperature monitoring, with a tolerance of ± 0.3 °C. Vital signs and tube patency were continuously monitored during thermal perfusion.

The experimental group (HIPEC Group) received 1 cycle of neoadjuvant laparoscopic hyperthermic intraperitoneal chemotherapy (paclitaxel 75 mg/m², 43 ± 0.3 °C, 400–500 mL/min, 60 min) followed by 3 cycles of paclitaxel + carboplatin, while the control group received only 3 cycles of intravenous neoadjuvant chemotherapy. In the HIPEC Group, an additional HIPEC was performed with the same chemotherapy regimen and dose, right after completing IDS.

#### Intravenous neoadjuvant chemotherapy (IV NACT)

Both the experimental and Control Group initiated intravenous chemotherapy as soon as possible (When patients have recovered well from surgery and can begin consuming a semi-liquid diet, typically within one week) after exploratory surgery, administering IV NACT every 3 weeks for a total of 3 cycles. The HIPEC Group received the following regimen: The first IV NACT followed by HIPEC: paclitaxel 100 mg/m² on day 1 and carboplatin AUC = 5 on day 2. The subsequent two regimens were: paclitaxel 175 mg/m² on day 1 and carboplatin AUC = 5 on day 2. The Control Group received only 3 cycles of intravenous chemotherapy, with paclitaxel 175 mg/m² on day 1 and carboplatin AUC = 5 on day 2. If patients in both groups experienced grade 4 adverse effects during chemotherapy, the subsequent dose of intravenous chemotherapy was reduced by 25% compared to the original dosage.

#### Interval debulking surgery (IDS)

After completing three cycles of chemotherapy, we conducted a disease assessment. IDS was carried out for cases of operable disease, while it was not considered if the disease had progressed during NACT. In our center, we take into account factors such as the KELIM score and radiological evaluation during MDT discussions to inform these decisions.

Open surgery under general anesthesia was performed, and the abdominal cavity was comprehensively investigated following a standardized pattern. FAGOTTI and PCI scores were determined based on the probe results. IDS was performed after exploration, to remove all visible lesions in the abdominal cavity.

At the time of IDS, the HIPEC Group underwent a second round of HIPEC before abdominal closure. This second HIPEC procedure was conducted in an open mode, with the abdominal cavity covered by plastic film for insulation. The regimen for the second HIPEC was identical to the initial procedure (paclitaxel 75 mg/m², normal saline 4000 ml, administered at a flow rate of 400–500 mL/min, and maintained at a temperature of 43 ± 0.3 °C for 60 min). Conversely, the Control Group did not undergo HIPEC.

Surgical outcomes, blood loss, and perioperative blood transfusion were recorded, and all excised specimens were sent for pathological evaluation.

Intravenous adjuvant chemotherapy (IV ACT): Both the HIPEC Group and the Control Group commenced intravenous adjuvant chemotherapy once patients had recovered well following IDS (typically within 2 weeks after surgery when they can tolerate a semi-liquid diet). The regimen for IV ACT mirrors that of IV NACT with 3 to 5 cycles (2 cycles initiated after CA125 levels normalize to < 25U/ml, with a minimum of 3 cycles and a maximum of 5 cycles). Similarly, patients experiencing grade 4 adverse effects during chemotherapy had their subsequent intravenous chemotherapy dose reduced by 25% compared to the original baseline.

Maintenance Treatment and Follow-up: All patients in this study were advised to undergo maintenance therapy following the completion of treatment in accordance with the NCCN guidelines [8]. Patients diagnosed with stage IIIC received PARP (poly ADP-ribose polymerase) inhibitor monotherapy. For stage IV patients, combination therapy with bevacizumab during adjuvant chemotherapy (15 mg/kg every 3 weeks regimen) was recommended 6 weeks after IDS, followed by maintenance therapy with both bevacizumab and PARP inhibitors after completing treatment. According to Chinese medical insurance policy, patients with Breast Cancer Susceptibility Genes (BRCA) mutation were prescribed Olapalil, while those without BRCA mutation were prescribed Nilapalil for maintenance therapy. Due to cost considerations (Olapalil being covered by medical insurance for first-line ovarian cancer maintenance treatment since 2020, Nilapalil since 2022, and bevacizumab since 2023), some early-enrolled patients did not accept the maintenance treatment. All patients underwent follow-up evaluations every 3 months for 2 years, and then every 6 months for the subsequent 3 years. Follow-up assessments included clinical examinations, serum Cancer Antigen 125 (CA 125) level monitoring, and enhanced Computed Tomography (CT). If recurrence was suspected, positron emission tomography-CT (PET-CT) imaging was performed.

### Outcomes

The initial primary endpoint of this randomized control trial was to compare differences in overall survival between the two groups. Secondary endpoints included the Progression-free survival, IDS R0 resection rate, the Aletti score of IDS, surgical safety (length of stay after laparoscopic exploration and IDS, length of days from IV NACT after laparoscopic exploration, intraoperative blood loss, perioperative red blood cell transfusion), surgical complications, and chemotherapy-related grade 3–4 adverse reactions (ARDs).

The ALETTI score is a scoring model proposed to assess the complexity of surgery based on the extent of surgical procedures [9]. This includes hysterectomy and bilateral salpingo-oophorectomy (1 point), omentectomy (1 point), pelvic lymph node resection (1 point), paraaortic lymph node resection (1 point), pelvic peritoneal dissection (1 point), abdominal peritoneal dissection (1 point), small intestine resection (1 point), liver resection (2 points), spleen resection (2 points), diaphragmatic dissection/resection (2 points), large intestine resection (2 points), direct sigmoid resection with end-to-end anastomosis (3 points). Surgery is considered complex if the ALETTI score is greater than 3. Chemotherapy-related adverse events were assessed using the Common Terminology Criteria for Adverse Events (CTCAE) version 3.0 [10].

For this interim analysis, we compared the adverse effects of chemotherapy and postoperative complication rates after IDS between the two groups. Additionally, we assessed the immediate treatment efficacy by comparing the rate of pathological chemotherapy response scores (CRS) after IDS.

CRS evaluates the pathological response to NACT in stage IIIC, IVA, and IVB HGSOC patients, primarily based on omentum lesion retraction after neoadjuvant chemotherapy [11]. CRS 1 indicates no or minimal tumor response, CRS 2 indicates a marked neoplastic response, and CRS 3 indicates a complete or near-complete response [11]. All patients underwent CRS scoring after IDS pathology confirmation by two pathologists.

### Sample size

The five-year overall survival (OS) rate for ovarian epithelial cancer patients treated at our center who did not undergo HIPEC was 38%. With a five-year survival rate of 38% in the control group and an HR of 0.53 for the HIPEC group, at a significance level (α) of 0.05 and power (1 - β) of 0.8, with an allocation ratio of 2:1, and accounting for an estimated dropout rate of 10%, we calculated the sample size for the HIPEC group to be 109 cases and for the control group to be 54 cases, resulting in a total sample size of 163 cases.

The data analysis cut-off time for this interim analysis was set after IDS for the 60th patient, who met the criteria for per-protocol (PP) analysis.

When planning this trial, we did not come across any studies involving taxane-based HIPEC. Therefore, we initially estimated the sample size for our study empirically. This number is consistent with the study conducted by Lim et al., where the total number of enrolled patients was 184. [12]. Nevertheless, we acknowledge that the sample size required for a large-scale, multi-center Phase III study may differ from our current number. This will depend on the statistical power set and the hazard ratio determined after obtaining the overall survival data for taxane-based HIPEC.

### Recruitment

In the recruitment process, any ovarian cancer patient admitted to the center who met the predefined inclusion criteria was eligible for consideration and potential recruitment. This approach confirms that there was no selection bias during the recruitment phase, as all eligible patients were considered. The screening of patients was conducted by skilled clinicians at the designated center, and the principal investigators assumed responsibility for evaluating the pretreatment assessments and making enrollment decisions, ensuring a rigorous and unbiased recruitment process.

### Randomization

In our study, we employed a straightforward randomization approach without employing blocks or stratifying factors. The randomization process was carried out using a pre-established code. The generation of the random allocation sequence was overseen by a statistician at the Clinical Research Center, which serves as the central body responsible for supervising all clinical trials. The generation of the randomized code was accomplished using the Random Number Generators within the SPSS statistical software, with the initial seed value set to a reproducible fixed value.

To maintain the integrity of the randomization, random numbers were placed inside sealed envelopes, each of which was sequentially numbered in accordance with the allocation sequence of the randomized numbers. These envelopes were subsequently opened in chronological order, corresponding to the admission sequence of the study subjects.

### Blinding

A blinded statistician assumed responsibility for the randomized assignment of interventions, either to the experimental group (HIPEC Group) or the Control Group. This assignment was executed through telephone contact or text messages, following the confirmation that the patient met the inclusion criteria and had provided informed consent. Importantly, both the patient and their caregivers were not blinded to the allocated intervention after the assignment. However, outcome assessments were meticulously conducted by pathologists who were strictly blinded to the intervention group.

### Statistical methods

For the analysis of the primary endpoint of this interim analysis (CRS rate), we employed the intention-to-treat (ITT) population, comprising all patients who were randomly assigned to a treatment group. Postoperative morbidity and mortality, on the other hand, were analyzed within the per-protocol (PP) population, which consisted of patients who underwent surgery following the completion of all planned treatment.

Our statistical analysis was carried out using Statistical Package for Social Science (SPSS) version 22.0 for Windows, provided by SPSS, Inc. based in Chicago, Illinois. The normality of the data was assessed using the Kolmogorov-Smirnov Test. Continuous data were described using the median and range, while categorical data were presented as frequencies and percentages. To compare differences in rates between the two groups, Fisher’s exact test was employed. All reported p-values are two-sided, and statistical significance was established at a threshold of less than 0.05. To ensure the robustness of our findings, all results underwent replication by two independent statisticians. Each statistician independently verified the results on two separate occasions, resulting in a total of four successful replications, thus enhancing the reliability of our statistical analyses.

## Results

**Fig. 2:**
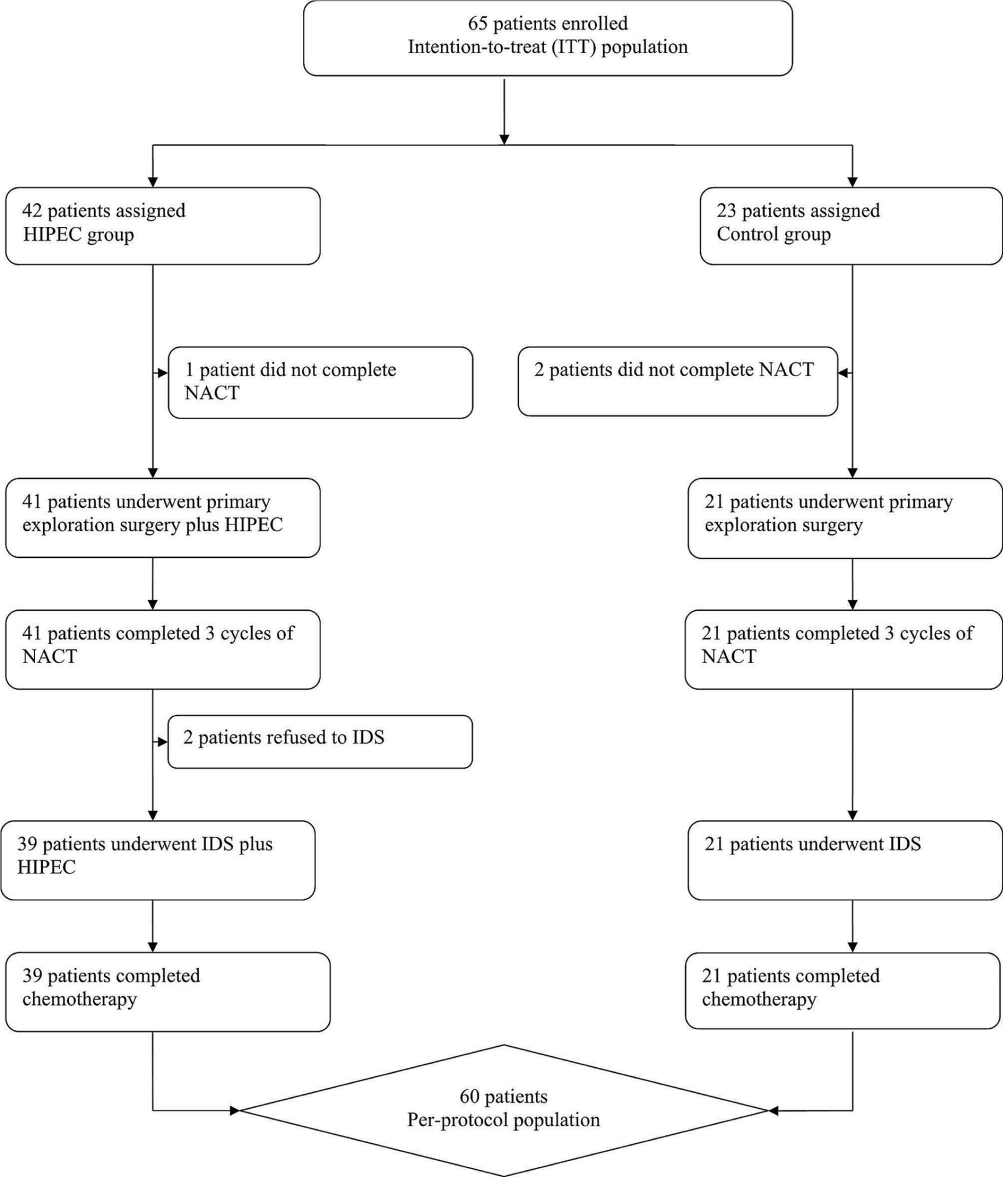
CONSORT diagram

This study commenced enrollment on September 2, 2019, and continued until March 3, 2023. A total of 65 patients enrolled, with 42 in the HIPEC Group and 23 in the Control Group. Of these, 60 patients were included in the final analysis. Five patients were excluded (3 from the HIPEC Group and 2 from the Control Group) for different reasons, including 3 patients not completing neoadjuvant chemotherapy as planned in our center, and 2 patients opting out of interval debulking surgery (Fig. 2). The final cohort comprised 39 cases in the HIPEC Group and 21 cases in the Control Group. The randomized control trial of this study is still ongoing; we present an interim analysis of partial data regarding the safety and immediate efficacy of both regimens. This analysis serves to determine the feasibility of proceeding with the planned trial.

All patients were confirmed to have High-Grade Serous Ovarian Carcinoma (HGSOC) through postoperative paraffin pathology, which was consistent with intraoperative rapid pathology assessment. Moreover, we did not observe any patients with progressive disease after three cycles of NACT. The baseline characteristics of the enrolled patients are detailed in Table 1. Grade 3 to 4 adverse effects related to NACT chemotherapy primarily involved hematological issues, with no significant differences observed between the two groups (Table 2).

**Table 1:** Demographic

Variables		Control Group	HIPEC Group	*P* value
Number of total cases		21	39	NA
Age (Years)	Median	56	65	0.068
BMI^1^	< 25 > 25	13(61.9) 8(38.1)	32(82.1) 7(17.9)	0.086
ECOG PS^2^	0	17(81.0)	31(79.5)	0.892
	1	4(19.0)	8(20.5)	
FIGO^3^ Stage	IIIC	8(38.1)	15(38.5)	1.000*
	IVA	0	1(2.6)	
	IVB	13(61.9)	23(59.0)	
Pretreatment CA 125^4^	Median	1541.2	1785.5	0.932
PCI^5^ Score (Exploration)	Median	18	18	0.549
PCI score (IDS)	Median	9	6	0.203
BRCA^6^ Mutation	None BRCA1	12(57.1) 4(19.0)	24(61.5) 9(23.1)	0.748*
	BRCA2	5(23.8)	6(15.4)	

*Fisher’s exact test

1. BMI: Body Mass Index

2. Eastern Cooperative Oncology Group Performance Status

3. Federation of Gynecology and Obstetrics

4. Cancer Antigen 125

5. Peritoneal Cancer Index scores

6. Breast Cancer Susceptibility Genes

**Table 2:** Treatment efficacy

Variables			Control Group		HIPEC Group			*P* value
Chemotherapy Response Score		1		7(33.3)		0	0.000*	
		2		13(61.9)		31(79.5)		
		3		1(4.8)		8(20.5)		
Aletti score for IDS^1^		≤ 3		8(38.1)		23(59.0)	0.136*	
		4–7		13(61.9)		14(35.9)		
		≥ 8		0		2(5.1)		
Residual tumor grad		R0		14(66.7)		27(69.2)	0.185*	
		R1		4(19.0)		11(28.2)		
		R2		3(14.3)		1(2.6)		
IDS blood loss(ml)		Median		400		300	0.412	
RBC^2^ transfusion(unit) in IDS		Median		2		3	0.598	
PHD^3^ after exploration		Median		6		5	0.039	
PHD after IDS		Median		11		9	0.076	
ICH^4^ after exploration		Median		4		3	0.157	
ICH after IDS		Median		12		7	0.016	
Grade 3–4 ADR^5^ of NACT		Yes		11(52.4)		16(41.0)	0.399	
		No		10(47.6)		23(59.0)		

*Fisher’s exact test

1. Alletti Score: Surgical complexity score

2. RBC: Red Blood Cell count

3. PHD: Postoperative Hospitalization Days

4. ICS: Interval (days) of Chemo start

5. ADR: adverse rate

There were no reoperations for postoperative complications or fatalities in either group. No statistically significant differences were observed between the two groups in ALETTI scores, perioperative blood loss during IDS, the requirement for perioperative red blood cell transfusions, the time from the first intravenous chemotherapy after laparoscopic exploration, or the length of hospital stay following IDS. However, the HIPEC Group did exhibit a shorter hospital stay following the initial laparoscopic exploration, as detailed in Table 2.

Complications associated with surgery included deep vein thrombosis of the lower limbs (DVT) in 1 case (HIPEC Group), pulmonary embolism in 1 case (Control Group), and postoperative non-infectious fever in 1 case (HIPEC Group). Postoperative intestinal obstruction occurred in 2 cases (1 in the HIPEC Group and 1 in the Control Group), postoperative non-infectious fever occurred in 1 case (HIPEC group), and postoperative hemorrhage exceeding 1000 ml occurred in 1 case (Control Group). All complications were successfully managed through conservative treatment.

**Fig. 3:**
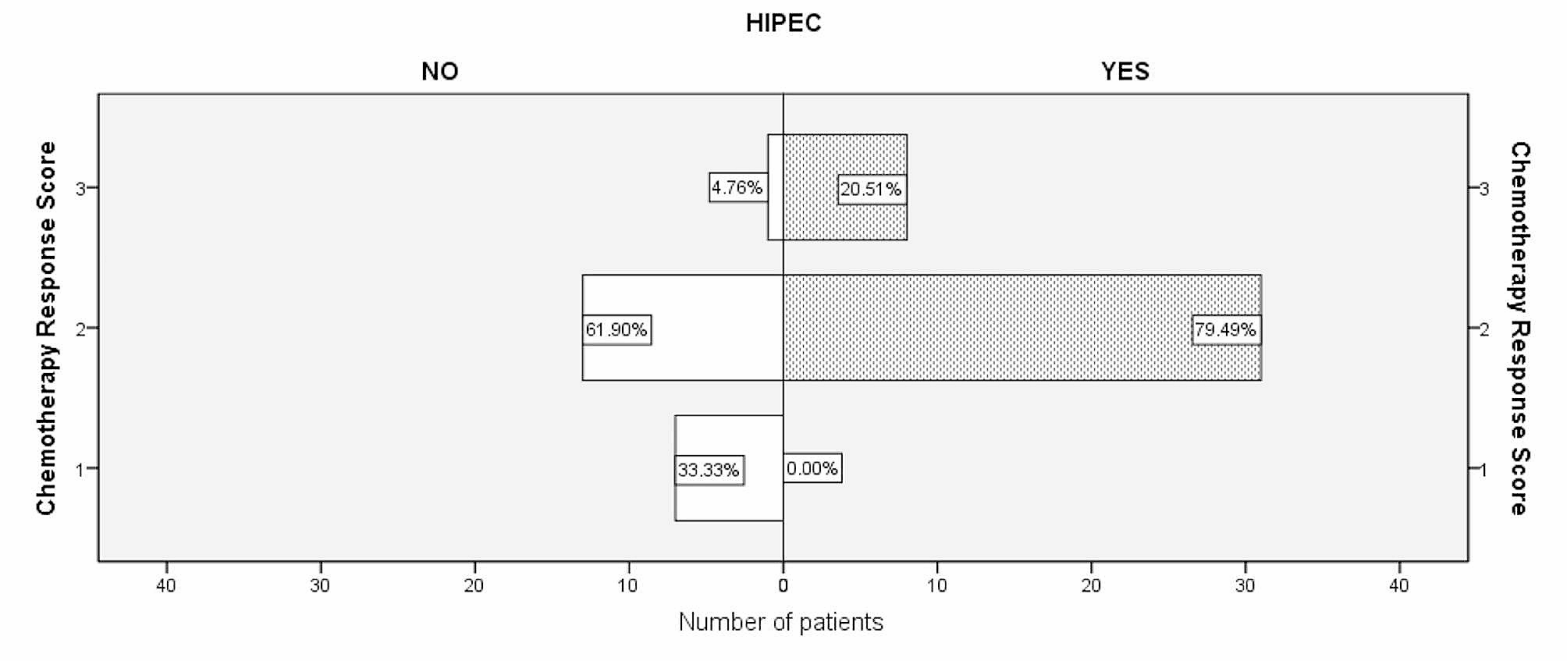
Difference in Chemotherapy Response Score

Among the 39 patients in the HIPEC Group, 31 cases achieved (79.49%) CRS2, and 6 cases (20.51%) achieved CRS3. In the Control Group consisting of 21 patients, 7 (33.33%) achieved CRS1, 14 (61.90%) achieved CRS2, and only 1 (4.76%) achieved CRS3. There was a statistically significant difference (*p* < 0.05) in the rate of CRS between the two groups (Fig. 3; Table 2). No significant difference was observed in R0 resection rates between the two groups. R2 resection was performed in 1 case (2.6%) in the HIPEC Group and 3 cases (14.3%) in the Control Group.

## Discussion

Currently, primary debulking surgery (PDS) combined with platinum-based chemotherapy stands as the primary treatment for High-Grade Serous Ovarian Carcinoma (HGSOC) [2, 8, 13]. However, in patients with advanced tumors, the extent of surgical intervention during PDS may be limited due to tumor spread, the need for extensive surgical procedures, and high surgical risks. This limitation often results in incomplete tumor cell reduction, leaving one or more residual lesions with a diameter exceeding 1 cm (classified as R2 residual disease).In such cases, interval debulking surgery(IDS) becomes an alternative option following neoadjuvant chemotherapy (NACT) [8, 13]. Although the NACT + IDS approach remains a subject of debate, studies have demonstrated that when IDS achieves complete tumor cell reduction (defined as R0 resection, indicating no visible disease), the survival outcomes can be comparable to those of optimal cell reduction surgery (defined asR1 resection, where one or more residual tumors measure ≤ 1 cm in diameter) performed during PDS [14, 15]. Moreover, the IDS + NACT model is associated with lower surgical risks, reduced complication rates, and enhanced quality of life for advanced ovarian cancer patients [14, 15]. To further improve the efficacy of NACT and minimize surgical risks, novel approaches are needed.

HyperthermicIntraperitonealChemotherapy (HIPEC) involves the continuous circulation of heated chemotherapy drugs within the abdominal cavity. Malignant tumors can experience irreversible damage at 43 °C for 1 h, while normal tissues can withstand temperatures up to 47 °C for the same duration. Compared to intravenous chemotherapy, peritoneal perfusion offers an advantage by bypassing the barrier effect of the peritoneum and directly and effectively targeting abdominal tumors [16, 17]. High temperatures cannot only directly damage tumor cells but also induce tumor cell apoptosis through proliferation, angiogenesis inhibition, and changes in cell membrane permeability [18]. The activation of heat shock proteins, especially when combined with paclitaxel, can enhance drug toxicity and promote tumor cell apoptosis [19]. Moreover, HIPEC has been shown to induce apoptosis in distant metastatic lesions.

In our study, we employed the Chemotherapy response scores (CRS) to assess the effectiveness of NACT. The CRS is recommended by the International Cancer Reporting Cooperation Organization and the European Society of Medical Oncology as a reliable prognostic tool for HGSOC patients [20, 21]. CRS3, in particular, has been associated with improved prognosis in HGSOC patients and can serve as an alternative indicator for Progression-Free Survival (PFS) [22]. While there was no difference in R0 resection rates between the two groups, the HIPEC Group exhibited a significantly reduced R2 resection rate. It’s worth noting that the assessment of tumor reduction largely relies on subjective evaluation by surgeons and may entail some margin for error. Notably, there were no instances of reoperation or mortality in either group, and there was no significant difference in the proportion of patients undergoing complex surgery (as indicated by an ALETTI score > 3) between the two groups. Therefore, paclitaxel-based HIPEC did not add complexity to IDS. Additionally, safety indicators such as length of hospital stay after IDS, time from intravenous NACT to laparoscopic exploration, intraoperative blood loss, and the incidence of perioperative red blood cell transfusion or grade 3–4 chemotherapy side effects did not significantly differ between the groups. Importantly, the HIPEC Group even demonstrated a shorter hospital stay after the initial laparoscopic exploration, suggesting that the addition of HIPEC had no adverse impact on patient outcomes.

No differences in the status of BRCA mutations were observed between the two groups. It is widely recognized that patients with BRCA mutations exhibit greater responsiveness to platinum-based drugs [23] and can benefit from maintenance therapy with PARP inhibitors [24, 25], often resulting in improved prognosis. Our findings suggest that the addition of HIPEC may offer greater benefits to patients with wild-type BRCA.

Our paclitaxel-based NLHIPEC approach offers several advantages. Firstly, it utilizes a minimally invasive closed-mode HIPEC, which confines treatment to a controlled space, minimizing heat loss, preventing drug evaporation, and reducing the risk of drug contamination. Pharmacokinetic studies of cisplatin HIPEC have demonstrated that minimally invasive HIPEC, when compared to open surgery, enhances drug uptake in peritoneal tissue, with potential correlations to improved survival [26].

Secondly, we opted for paclitaxel HIPEC instead of platinum-based drugs, marking an intriguing innovation point. Paclitaxel isn’t commonly utilized in HIPEC, as most ongoing HIPEC studies focus on platinum-based drugs. This trend may stem from several reasons: (1) a historical belief that paclitaxel lacks thermal synergistic effects, with inconsistent data regarding the thermal enhancement of paclitaxel cytotoxicity [27]; (2) Paclitaxel operates on a cell cycle-specific basis, exerting its anti-tumor effect by impeding mitosis [28, 29]. It’s effective only during the mitotic phase of cell division, while for HIPEC, an ideal drug is preferably cell cycle non-specific since it’s a singular treatment. However, studies involving mice [30] or human breast cancer patients [31]suggest that paclitaxel-induced mitotic cessation doesn’t necessarily correlate with tumor response. These studies imply that paclitaxel’s anti-tumor effects extend beyond mitosis, with the efficacy also linked to paclitaxel-induced apoptosis and baseline apoptosis. Furthermore, a new perspective has emerged in recent years suggesting that paclitaxel enhances the sensitivity of SKOV3 cells to hyperthermia by inhibiting heat shock protein 27, showcasing a synergistic effect between paclitaxel and hyperthermia at the cellular level [19].

Paclitaxel boasts its own unique advantages, primarily in terms of pharmacokinetics. Compared to carboplatin (at 10:1) or cisplatin (at 20:1), paclitaxel exhibits a higher intraperitoneal-to-intravenous concentration ratio (AUC), resulting in prolonged abdominal retention time and increased local concentrations. One study revealed that the maximum intra-abdominal drug concentration during HIPEC was 12–30 times higher than the maximum blood drug concentration after intravenous chemotherapy [32]. Despite this, there isn’t an authoritative report on the ideal paclitaxel dosage. However, based on the aforementioned scenario and in conjunction with findings from a phase I experiment [33], which indicated a significant increase in adverse reactions with doses reaching 175 mg/m^2 and above, we opted against using the conventional intravenous dose (175 mg/m^2). Instead, we chose a reduced dose (75 mg/m^2). In the HIPEC Group, intravenous chemotherapy followed by HIPEC involved paclitaxel at 100 mg/m^2 on Day 1. The rationale behind reducing the dose of paclitaxel here stemmed from our desire to maintain the total dose consistent across both groups during the study’s design phase. This approach allowed for a comparison between the two groups under equivalent exposure to chemotherapy drugs, facilitating an assessment of the effectiveness and safety of HIPEC. Nonetheless, we acknowledge that the effectiveness of phase-specific chemotherapy drugs is influenced by total dosage and timing. Dividing the same dosage may not be equivalent to a single administration. Due to limited reports on paclitaxel-based HIPEC, we selected a relatively safe dosage.

Another advantage of paclitaxel is its lower toxicity compared to platinum drugs. This is due to several factors: firstly, paclitaxel doesn’t induce renal toxicity, a concern observed with OVHIPEC01. Secondly, its high molecular weight (853.9 g/mol) contrasts with cisplatin (300.01 g/mol) [34], making it less easily absorbed through the peritoneum and consequently reducing systemic toxic effects. While paclitaxel isn’t a mainstream drug for HIPEC, there have been relevant reports with small sample sizes in the past. Studies consolidating paclitaxel HIPEC for ovarian cancer have shown promising recurrence and survival rates [35]. When compared with carboplatin HIPEC for consolidation therapy, paclitaxel HIPEC demonstrated a tendency towards better survival rates with fewer toxic effects [36]. Recent clinical trials have also indicated that paclitaxel HIPEC surpasses cisplatin and carboplatin in terms of Progression-Free Survival (PFS) and Overall Survival (OS) [37, 38]. Notably, paclitaxel HIPEC is currently being investigated in the HIPECOVA trial (NCT02681432) for advanced and recurrent ovarian cancer, with positive preliminary results [39].

Lastly, our study maintained a treatment temperature of 43°C, with strict temperature control provided by a body cavity hyperthermic perfusion therapy system. Research has shown thattemperaturesrangingfrom41-43°Cselectivelydestroycancercellswithout adversely affecting normal tissues [40, 41]. Studies on paclitaxel HIPEC have demonstrated that temperatures below 43 °C enhance paclitaxel’s pro-apoptotic effects, with temperatures exceeding 43 °C offering no significant additional benefit, confirming 43 °C as the optimal and safe temperature. In our study, HIPEC was administered once under anesthesia supervision during surgery, reducing risks during perfusion therapy, as well as minimizing catheter-related complications and uneven drug distribution due to postoperative adhesions.

Our study has certain limitations. First, as preliminary data analysis from a Randomized control trial with a small sample size, we have not yet conducted long-term follow-ups for patients regarding Progression-FreeSurvival (PFS)and Overal lSurvival(OS). Additionally, due to economic constraints, our study included only the BRCA status of patients and did not account for Homologous Recombination Deficiency (HRD) status. Consequently, there may be a lack of efficacy evaluation for patients with wild-type BRCA and mutations.

## Conclusion

Our study has provided evidence that the sequential administration of paclitaxel-based Hyperthermic Intraperitoneal Chemotherapy (HIPEC) followed by intravenous neoadjuvant chemotherapy is associated with enhanced tumor response to neoadjuvant chemotherapy. Furthermore, this approach holds promise for improving Progression-Free Survival (PFS) while simplifying Interval Debulking Surgery (IDS). Importantly, the inclusion of HIPEC did not adversely affect the efficacy of NACT or the surgical procedures. Further research, including larger multi-center clinical trials and longer-term follow-ups for survival rate, is warranted to validate and expand upon these findings.

## Data Availability

Datasets utilized and analyzed during this study are available from the corresponding author upon reasonable request.
